# A Deadenylase Assay by Size-Exclusion Chromatography

**DOI:** 10.1371/journal.pone.0033700

**Published:** 2012-03-19

**Authors:** Guang-Jun He, Yong-Bin Yan

**Affiliations:** State Key Laboratory of Biomembrane and Membrane Biotechnology, School of Life Sciences, Tsinghua University, Beijing, China; University of Cambridge, United Kingdom

## Abstract

The shortening of the 3′-end poly(A) tail, also called deadenylation, is crucial to the regulation of mRNA processing, transportation, translation and degradation. The deadenylation process is achieved by deadenylases, which specifically catalyze the removal of the poly(A) tail at the 3′-end of eukaryotic mRNAs and release 5′-AMP as the product. To achieve their physiological functions, all deadenylases have numerous binding partners that may regulate their catalytic properties or recruit them into various protein complexes. To study the effects of various partners, it is important to develop new deadenylase assay that can be applied either *in vivo* or *in vitro*. In this research, we developed the deadenylase assay by the size-exclusion chromatography (SEC) method. The SEC analysis indicated that the poly(A) or oligo(A) substrate and the product AMP could be successfully separated and quantified. The enzymatic parameters of deadenylase could be obtained by quantifying the AMP generation. When using the commercial poly(A) as the substrate, a biphasic catalytic process was observed, which might correlate to the two distinct states of poly(A) in the commercial samples. Different lots of commercial poly(A) had dissimilar size distributions and were dissimilar in response to the degradation of deadenylase. The deadenylation pattern, processive or distributive, could also be investigated using the SEC assay by monitoring the status of the substrate and the generation kinetics of AMP and A2. The SEC assay was applicable to both simple samples using the purified enzyme and complex enzyme reaction conditions such as using protein mixtures or crude cell extracts as samples. The influence of solutes with absorption at 254 nm could be successfully eliminated by constructing the different SEC profiles.

## Introduction

Almost all mature eukaryotic mRNAs contain a 5′ cap structure and a poly(A) tail at the 3′-end, and these non-coding elements are crucial to the regulation of mRNA processing, transportation, translation and degradation [1]–[4]. The shortening of the 3′-end poly(A) tail, also called deadenylation, often leads to translational repression and is the rate-limited step of eukaryotic mRNA decay [3]–[6]. The deadenylation process is achieved by deadenylases, which specifically catalyze the removal of the poly(A) tail at the 3′-end of eukaryotic mRNAs. Several deadenylases conserved in most eukaryotes have been characterized, including three widely studied enzymes Pan2-Pan3, Ccr4-Pop2 and poly(A)-specific ribonuclease (PARN) [6], [7]. Due to the importance of the regulation of mRNA fate, these deadenylases have been found to play a crucial role in the regulation of diverse physiological processes including cell cycle, embryonic development and stress response in both the animal and plant kingdom [8]–[11].

The deadenylases characterized thus far can be divided into two groups: the DEDD and EEP superfamilies. Although all deadenylases catalyze the same reaction of hydrolyzing the poly(A) tail at the 3′-end with the requirement of a free 3′ hydroxyl group and releasing 5′-AMP as the product, they have diverse functions by participating into various complexes and distinct catalytic properties partially determined by their domain organizations. For example, PARN is unique by its catalytic efficiency [12], highly processive and allosteric catalysis stimulated by the existence of the 5′-cap structure [13]–[18]. All deadenylases have numerous binding partners that may regulate their catalytic properties or recruit them into various protein complexes. To speculate the effects of various partners, it is important to develop new deadenylase assay that can be applied either *in vivo* or *in vitro*.

Currently, two kinds of method are used for deadenylase assay. One is based on radioactive isotopic or fluorescein-isothiocyanate labeled RNA substrates [19]–[23]. In this method, ^32^P-labeled or commercially synthesized 5′-fluorescent dye-labeled RNA substrates are used for the enzymatic reaction. After reaction, the samples are fractionated on a polyacrylamide denaturing gel containing urea. Deadenylase activity is obtained by detecting and quantifying the released radioactive mononucleotides. This method has been widely used in literature due to its high sensitivity although it is radioactive or expensive, laborious and time-consuming. The other method utilizes the commercially available poly(A) as the substrate and is based on the methylene blue colorimetry [24]. The methylene blue molecules can insert into the polynucleotide chain, and the insertion results in a shift in the absorbance maximum in the UV spectrum [25]. This method is nonradioactive, easily performed and less time-consuming. However, the accuracy of this method is significantly affected by the status of the substrate such as the molecular size distribution of the commercially available poly(A) since the methylene blue method monitors the amounts of the long substrates. The methylene blue method is not applicable for short oligoadenylic acid [oligo(A)] as the substrate since short substrate has a relative low methylene blue binding affinity.

Size-exclusion chromatography (SEC), also called gel filtration or gel permeation chromatography, is one of the most widely used method to separate or identify molecules with different molecular sizes or shapes. In this research, we found that the synthesized oligo(A) could be successfully separated by SEC, which suggested SEC or the other chromatography methods could be used to determine the enzymatic properties of exonucleases. By using PARN as a model enzyme, the deadenylase assay by SEC was successfully developed. Particularly, the SEC method was found to be able to determine whether the enzyme follows a processive or distributive manner in catalysis.

## Materials and Methods

### Materials

Tris and adenosine monophosphate (AMP) were purchased from AMRESCO. Imidazole, methylene blue and polyadenylic acid potassium salt were obtained from Sigma-Aldrich, Inc. Dithiothreitol (DTT) and isopropyl-1-thio-β-D-galactopyranoside (IPTG) were purchased from Promega. The 2-, 6-and 20-mer oligo(A) (A2, A6, A20) without the 5′ phosphate group and 20-mer oligo(dA) were synthesized by TaKaRa Biotechnology Co., Ltd (Dalian, China). All other chemicals were local products of analytical grade.

### Protein expression, purification and sample preparation

The plasmid for the wild type human PARN (p74) was kindly provided by Professor Anders Virtanen (Uppsala University, Sweden). The truncated mutant p60 (residues 1–520) with the removal of the C-terminal domain was constructed by site-directed mutagenesis using the following primers: forward, 5′-CGATGTCACATATGGAGATAATCAGGAGC-3′, reverse 5′-GATCCTCGAGCTACTTCTCTTCCTGTTTTC-3′. The gene was cloned to the vector pET-28a (Novagen) and verified by sequencing. The protein was overexpressed in *Escherichia coli* BL21 (DE3) (Stratagene, Heidelberg, Germany) and purified as described previously with some modifications [26], [27]. In brief, the proteins were purified by Ni^2+^ affinity chromatography (GE Healthcare), and the final products were collected from a Superdex 200 10/30 GL column equipped on an ÄKTA purifier (GE Healthcare). The purity of the final products was above 98% as estimated by SDS-PAGE and SEC analysis. The protein concentration was determined according to the Bradford method [28] using bovine serum albumin as a standard. The proteins for the experiments in this research were dissolved in 20 mM Tris-HCl, pH 8.0, 100 mM KCl, 0.5 mM DTT, 0.2 mM EDTA and 10% (v/v) glycerol].

### Deadenylase assay by methylene blue colorimetry

The enzymatic activity was measured according to the standard methylene blue method as described previously [24] with some modifications. In brief, methylene blue stock solutions was prepared by dissolving 1.2 mg methylene blue in 100 ml Mops buffer (100 mM Mops-KOH, 2 mM EDTA, pH 7.5) and the absorbance at 688 nm was adjusted to 0.6±1%. The standard reaction buffer was buffer A (20 mM Tris-HCl, pH 7.0, 100 mM KCl, 0.5 mM DTT, 0.2 mM EDTA and 10% (v/v) glycerol]) with the addition of 1.5 mM MgCl_2_. The substrate A200 was dissolved in the reaction buffer with the concentration of 100 µg/ml. The reaction was initiated by mixing 10 µl enzyme and 40 µl of A200 in the standard reaction buffer. After 8 min reaction at 30°C, methylene blue buffer was added to terminate the reaction. Then the solution was incubated for another 15 min in the dark, and the absorbance at 662 nm was measured using an Ultraspec 4300 pro UV/Visible spectrophotometer.

### Deadenylase assay by SEC

SEC experiments were performed on an ÄKTA purifier equipped with a Superdex 200 10/30 GL column (GE Healthcare). The column was pre-equilibrated for 2 column volumes until the UV absorbance and conductance lines were at the same level as that of the control (buffer A). The RNA substrate (A20, A6 and A2) or DNA substrate (dA20) was dissolved in buffer A with the addition of 1.5 mM MgCl_2_ and quantified according to the instructions from the manufacture. The reaction was initiated by mixing 20 µl enzyme and 100 µl substrate stock solutions. After incubated at 37°C for a given time, the samples were cooled on ice to quench the reaction and EDTA was added when necessary. Then the samples were loaded on the injection ring with the volume of 100 µl. The absorbance at 280 nm, 254 nm and 215 nm were monitored simultaneously. The peak area of AMP was calculated using the software Origin (OriginLab Corp.) and the concentration of AMP was calculated thereby according to the standard curve. The enzymatic data were fitted using the Michaelis–Menten equation to obtain the kinetic constants *K*_m_ and *V*_max_.

### Cell culture and extraction

The human embryonic kidney (HEK)-293T cell line was obtained from the American Type Culture Collection (ATCC, Manassas, VA). The HEK-293T cells were maintained in DMEM (Dulbecco's modified Eagle's medium; Gibco) with 10% FBS (fetal bovine serum; Gibco). Cells were cultured at 37°C in a humidified incubator. The coding sequence of the full-length PARN was subcloned into pcDNA3.1 (Invitrogen) containing a Flag tag at the N-terminus. Prior to transfection, HEK-293T cells were seeded in a 60-mm dish for 24 h. The plasmids were transfected using the Vigofect transfection reagent (Vigorous) according to the manufacturer's instructions. After 20–24 h transfection, the HEK-293T cells were washed with the ice-cold PBS buffer, harvested, and stored at −80°C. The cell lysis buffer was buffer A with the addition of 1 mM PMSF and 1 mg/ml leupeptin. The cell lysates were centrifuged at 15000 g for 30 min at 4°C, and the supernatant was used for the enzyme assay.

### Spectroscopy

Circular dichroism (CD) spectra were recorded on a Jasco-715 spectrophotometer using a cell with a path length of 0.1 cm. UV absorption spectra were measured on an Ultraspec 4300 pro UV/Visible spectrophotometer.

## Results and Discussion

### Characterization of the substrates and AMP

**Figure 1:**
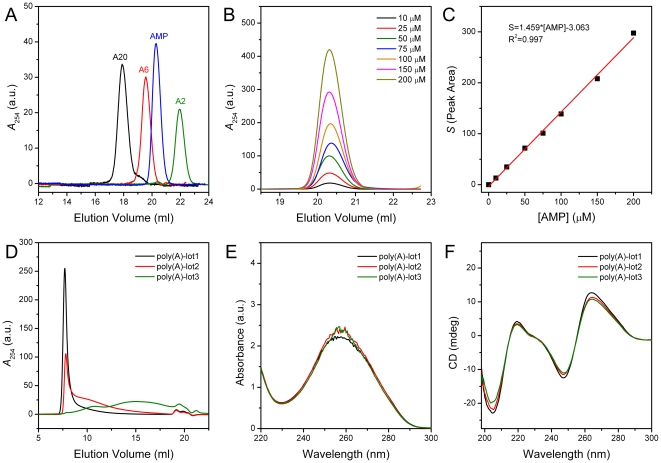
Characterization of the synthetic oligo(A) and commercial poly(A) samples. (A) SEC profiles of A20, A6, A2 and AMP. The concentration of A20, A6, A2 and AMP were 2, 5, 10 and 25 µM, respectively. (B) SEC profiles of AMP with various concentrations. (C) Relationship between the peak area of the AMP elution peak and the AMP concentration. The raw data is shown as square symbols and the fitted curve is presented as a solid line. (D) SEC profiles of three batches of commercial poly(A) samples with a mass concentration of 100 µg/ml. (E) UV absorption spectra of the three batches of commercial poly(A) samples with a concentration of 100 µg/ml. The UV absorption spectra were measured by Ultraspec 4300 pro UV/Visible spectrophotometer using a cell with a path length of 1 cm. (F) CD spectra of 100 µg/ml poly(A) samples. The CD spectra were recorded on a Jasco-715 spectrophotometer using a cell with a path length of 0.1 cm. All samples were prepared in buffer A. The SEC analysis was performed using a Superdex 200 10/30 GL column, and the absorbance at 254 nm was monitored.

As a 3′-exoribonuclease which specifically catalyze the degradation of the mRNA poly(A) tail, the product of deadenylase catalysis is 5′-AMP. For in vitro deadenylase assay, synthetic oligo(A) or commercial available poly(A) are frequently used as the substrate [19], [20], [23], [24], [29]. Since the product and the substrate are significantly different in their molecular size, it is possible to separate them by techniques such as SEC that could recognize the size of the samples. As presented in Figure 1A, the elution volume of A20, A6, A2 and AMP were around 18.0, 19.6, 22.0 and 20.3 ml, respectively. The elution volume increased with the decrease of molecular weight except for that the elution volume of A2 is larger than that of AMP. This may be caused by that the substrates A20, A6 and A2 were synthetic oligo(A) without the 5′ phosphate group, which means that the charge of A2 was −1 while that of AMP was −2. For small molecules such as A2 and AMP, the SEC elution volume was affected by both the molecular weight and the charge of the molecule. It is worth noting that there were some overlap between the elution peaks of A6 and AMP on the Superdex 200 column, and the usage of the other column may be better when using A6 as the substrate of deadenylase. Nonetheless, the result in Figure 1A indicated that SEC was able to separate the oligo(A) substrate and product of deadenylase. In order to quantify the AMP concentration from the elution peak, standard curve was determined by plotting the AMP concentration vs. the peak area in the SEC profile (Figure 1B and 1C), and a good linear relationship was observed. It is worth noting that the height of the peak also showed a good linear relationship (data not shown), revealing that both the peak area and height could be used for quantitative measurement. In addition to AMP, the standard curves of the substrates could also be determined using the same method (data not shown). To minimize the system error of the assay, the volume of prepared samples were 120 µl and the load volume was fixed to 100 µl.

Commercial poly(A) were also frequently used in the *in vitro* assay for deadenylases. According to the instructions from the manufacture, poly(A) were prepared from ADP with polynucleotide phosphorylase, and the number of adenosine ranged from 400–6000. The wide size distribution of the commercial poly(A) makes it impossible to determine the molecular weight and the molar concentration of poly(A), while only the mass concentration can be determined. Three batches of poly(A) with different production lot and storage time were characterized and used for the assay to check whether the different lots of products affected the enzymatic parameter determination. As shown in Figure 1D, the three lots of poly(A) samples were distinct in size distributions. Among them, the elution peak of poly(A)-lot1 was the sharpest with an elution volume of about 8.0 ml, which is close to the void volume of the column. The elution profile of poly(A)-lot3 showed a smeared peak, while that of poly(A)-lot2 was between those of poly(A)-lot1 and poly(A)-lot3. Although significant discrepancy was observed for the elution profiles, the peak areas of the three lots of poly(A) substrates were approximately equivalent with a variation within 5%, confirming that the same mass concentration of poly(A) was used for the SEC analysis. UV absorbance and CD spectroscopy were measured to further investigate the properties of the three lots of commercial poly(A). As shown in Figure 1E and 1F, no significant difference was observed for the three lots of samples. The slight discrepancy in the CD spectra might be caused by the dissimilar size distribution or secondary structure of the three samples. These results suggested that the major difference among the three patches of poly(A) preparations was the size distribution of the poly(A) molecules.

### Deadenylase assay by SEC using A20 as the substrate

**Figure 2:**
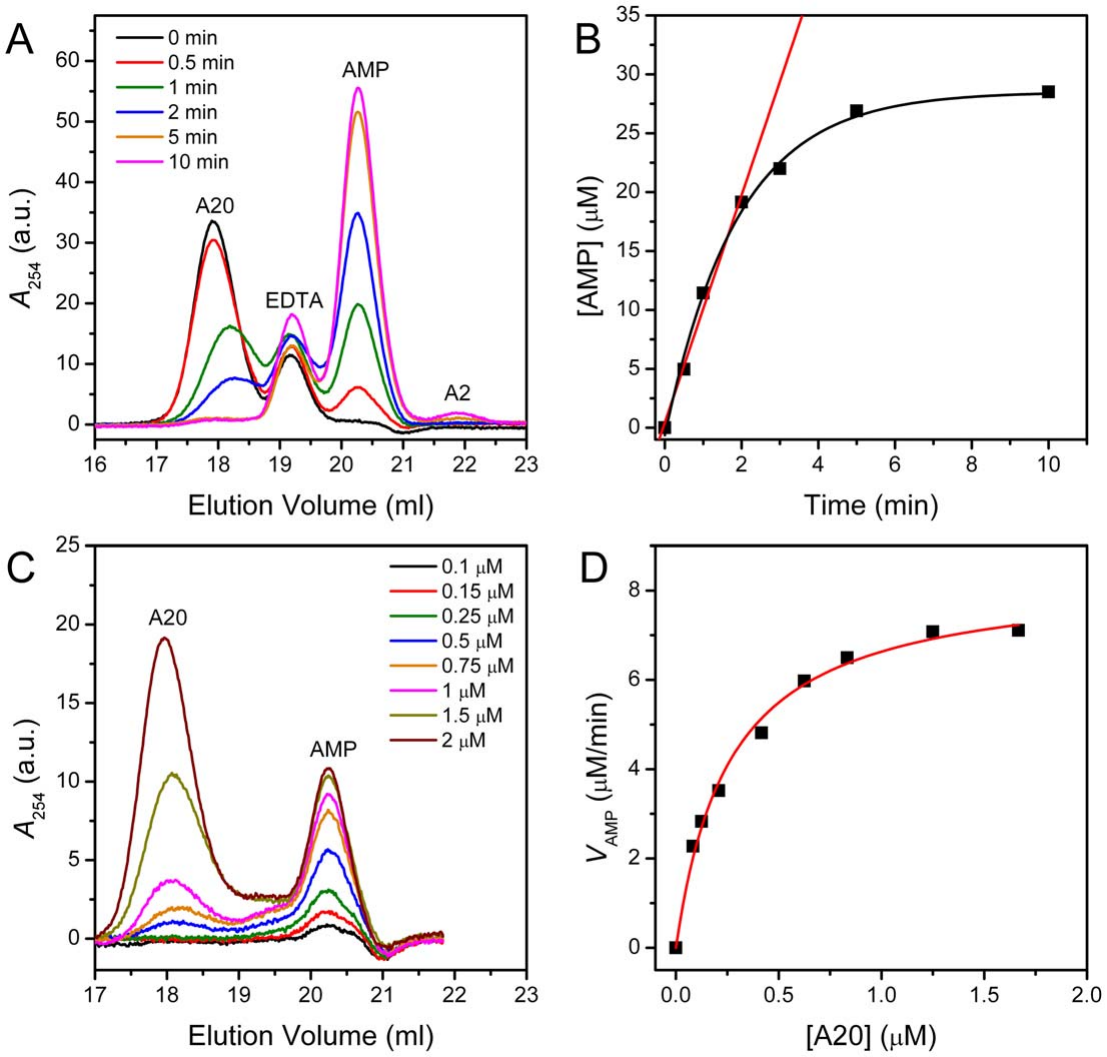
SEC assay of PARN using A20 as the substrate. (A) The elution profiles of the reaction solutions quenched at different time. The initial A20 concentration was 2 µM, and the concentration of PARN was 0.001 mg/ml. The reaction was initiated by mixing 20 µl enzyme and 100 µl substrate. After incubated at 37°C for a given time, the samples were cooled on ice. EDTA was added to the samples to terminate the reaction, and then the samples were injected into the SEC column. (B) Time-course changes of the AMP during reaction. The concentration of AMP was calculated from the peak area using the standard curve shown in Figure 1C. (C) SEC profiles of the reaction solutions using various concentrations of A20 as the substrate. The A20 concentration was ranged from 0 to 2 µM and the final concentration of PARN was 0.001 mg/ml. The reaction time was 1 min. (D) Determination of the enzymatic parameters using the Michaelis–Menten kinetics. The fitted curve is presented as a solid line.

**Figure 3:**
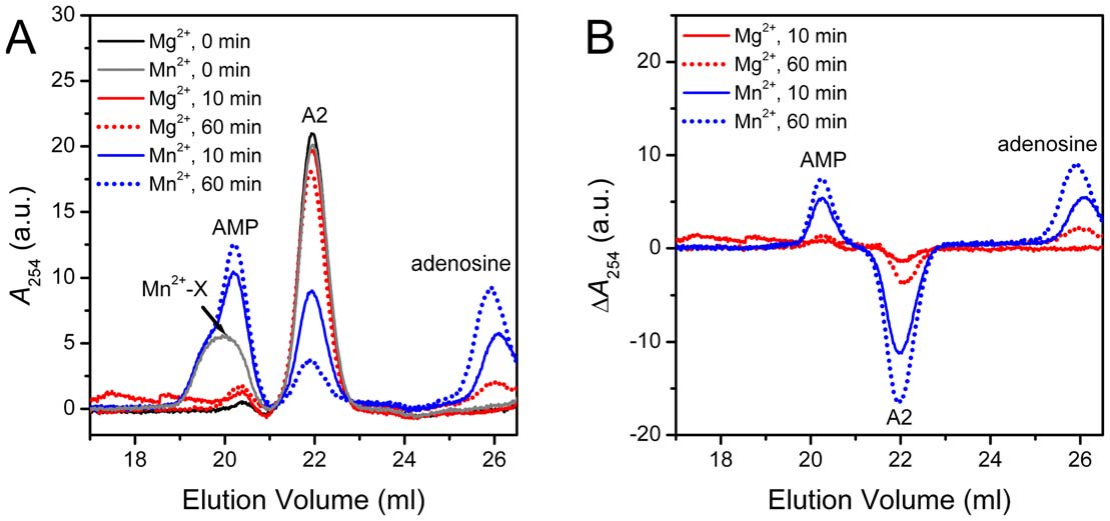
Degradation of A2 by PARN in the presence of Mg^2+^ or Mn^2+^. (A) The elution profiles of the reaction solutions containing Mg^2+^ or Mn^2+^ quenched at different time. The concentrations of A2, PARN and the divalent mental ions were 10 µM, 0.02 mg/ml and 3 mM, respectively. The peak appeared in the buffer containing Mn^2+^ at about 20 ml was from Mn^2+^-coordinated molecules (labeled as Mn^2+^-X), and this peak maintained unchanged during the reaction. To avoid the influence of this peak, difference profiles are obtained and shown in panel B. Since the synthetic A2 contained no 5′-phosphate group, the degradation of A2 resulted in the production of 5′-AMP and adenosine. (B) The difference profiles obtained by subtracting the SEC profile recorded at a given time by that at 0 min.

The data in Figure 1A showed that the elution peak of A20 and A2 were well separated from the AMP peak, indicating that both of them were suitable substrates for the deadenylase assay by SEC method. Due to the resolution of the Superdex 200 column, the peak of oligo(A) smaller than A6 overlapped with that of AMP and column with better resolution should be chosen. Considering that the wild type PARN usually underwent proteolysis after storage, the mutant p60 with the removal of the C-terminal domain of the wild type protein was used in this research. To optimize the SEC assay using A20 as the substrate, the dependence of PARN catalysis was studied on the concentrations of A20 and enzyme as well as reaction time. As shown in Figure 2, as the reaction proceeded, the peak of A20 decreased accompanied with a shift to the larger elution volume after 1 min reaction, while the peak area of AMP increased thereby. An additional peak appeared at about 19.2 ml, which remained unchanged along with the reaction time. This elution peak was characterized as EDTA, which was added to the reaction solutions to terminate the reaction. To avoid the influence of the EDTA peak to the elution profile, the difference profiles are recommended to be used instead of the original spectra (data not shown, please refer to Figure 3B). In this work, we also tested the method of quenching the reaction on ice but not the addition of EDTA, and it is also applicable since the quenched samples were greatly diluted in the SEC column with buffers containing no divalent metal ions (Figure 2C). A small peak at 22.0 ml appeared after 10 min reaction, which was characterized to be A2. The appearance of A2 also confirmed that PARN is a 3′-5′ exonuclease [19], [20]. The production of AMP increased linearly with the time within 2 min reaction, followed by decrease in the production rate.

The enzyme activity was obtained by the initial velocity of the enzymatic reaction in which the enzyme was saturated by the substrates. It is worth noting that the appropriate concentration of the enzyme and substrates as well as the reaction time is important for discontinuous assay to ensure that the amount of products is within the linear range of the velocity. Under our experimental conditions, when the concentration of A20 and p60 were 2 µM and 0.001 mg/ml, respectively, the AMP was produced linearly to about 20 µM within 2 min. The enzymatic parameters of p60 were determined by varying the concentration of A20 from 0.1 to 2 µM with a p60 concentration of 0.001 mg/ml (16.08 nM) and a reaction time of 1 min. Although PARN is an allosteric enzyme, it has been shown that at physiological KCl concentrations, the catalysis of PARN follows the Michaelis–Menten formulation [17]. Thus the initial velocity data at various A20 concentration were fitted by the classical Michaelis–Menten equation (Figure 2D), which revealed that the *K*_m_-A20, *V*_max_-A20 and *k*_cat_ values of p60 were 0.26±0.02 µM, 8.4±0.2 µM/min and 8.7 s^−1^, respectively.

### Deadenylase assay by SEC using A2 as the substrate

The previous study by Ren et al. has shown that A2 can hardly be hydrolyzed by Mg^2+^-coordinated PARN, but can by Mn^2+^, Zn^2+^ or Co^2+^-coordinated enzyme [30]. For the native Mg^2+^-coordinated PARN, A3 is the shortest oligo(A) substrate. Thus A2 was used as the substrate to check the validation of the deadenylase assay by the SEC method. As shown in Figure 3, in the presence of 3 mM Mg^2+^, A2 was hardly hydrolyzed when compared to A20 (Figure 2) even though a high enzyme concentration of 0.02 mg/ml was used. After 60 min reaction, a small amount of AMP was produced. In the SEC profile shown in Figure 3A, a new peak with an elution volume of 26.0 ml appeared. This new peak was subsequently characterized as adenosine since the synthetic A2 lacking the 5′ phosphate group will hydrolyze to AMP and adenosine. Consistent with the previous observation [30], A2 could be efficiently degraded in the presence of Mn^2+^. According to the AMP production, the enzyme activity in the presence of Mn^2+^ was estimated to be more than 80 times higher than that in the presence of Mg^2+^. Moreover, the Mn^2+^-coordinated molecules also have absorption at 254 nm, and the construction of difference profiles by subtraction of the control can successfully eliminate the influence of the solutes with absorption bands in the SEC profiles (Figure 3B).

### Deadenylase assay by SEC using commercial poly(A) as the substrate

**Figure 4:**
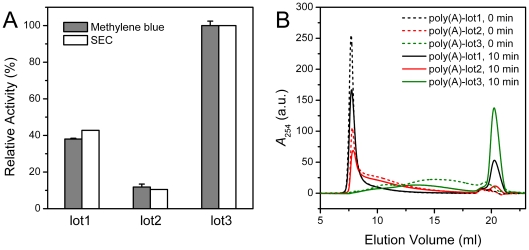
Relative activities of PARN when using the three lots of commercial poly(A) as the substrate. (A) Activities determined by the methylene blue and SEC assay. The mass concentration of poly(A) were all 100 µg/ml. The final concentration of enzyme was 0.04 mg/ml, and the reaction time was 10 min. (B) SEC profiles of the reaction solutions quenched at 0 and 10 min using the three lots of poly(A) as the substrate.

As mentioned above, three batches of poly(A) with different production lots were found to be distinct in their size distribution as characterized by SEC analysis. First of all, the methylene blue assay was used to determine the enzymatic activity of the three samples. As shown in Figure 4A, the enzymatic activity differed dramatically when different lots of commercial poly(A) were used as the substrates. Almost ten-fold difference was observed for the highest and lowest activity. It is not surprising that the methylene blue assay will be affected by the size distributions of the poly(A) substrate since the methylene blue molecules bind more tightly with the long chain poly(A). To speculate whether the activity measured by the methylene blue assay was affected by the size distributions of the substrates, the reaction samples were analyzed by SEC after reacted for the same time as that used in the methylene blue assay, and the elution profiles are presented in Figure 4B. The amounts of AMP production with different substrates coincided with the enzymatic activity measured by the methylene blue assay (Figure 4A). This suggested that the discrepancy in the measured activity was not simply determined by the size distribution of the substrate.

**Figure 5:**
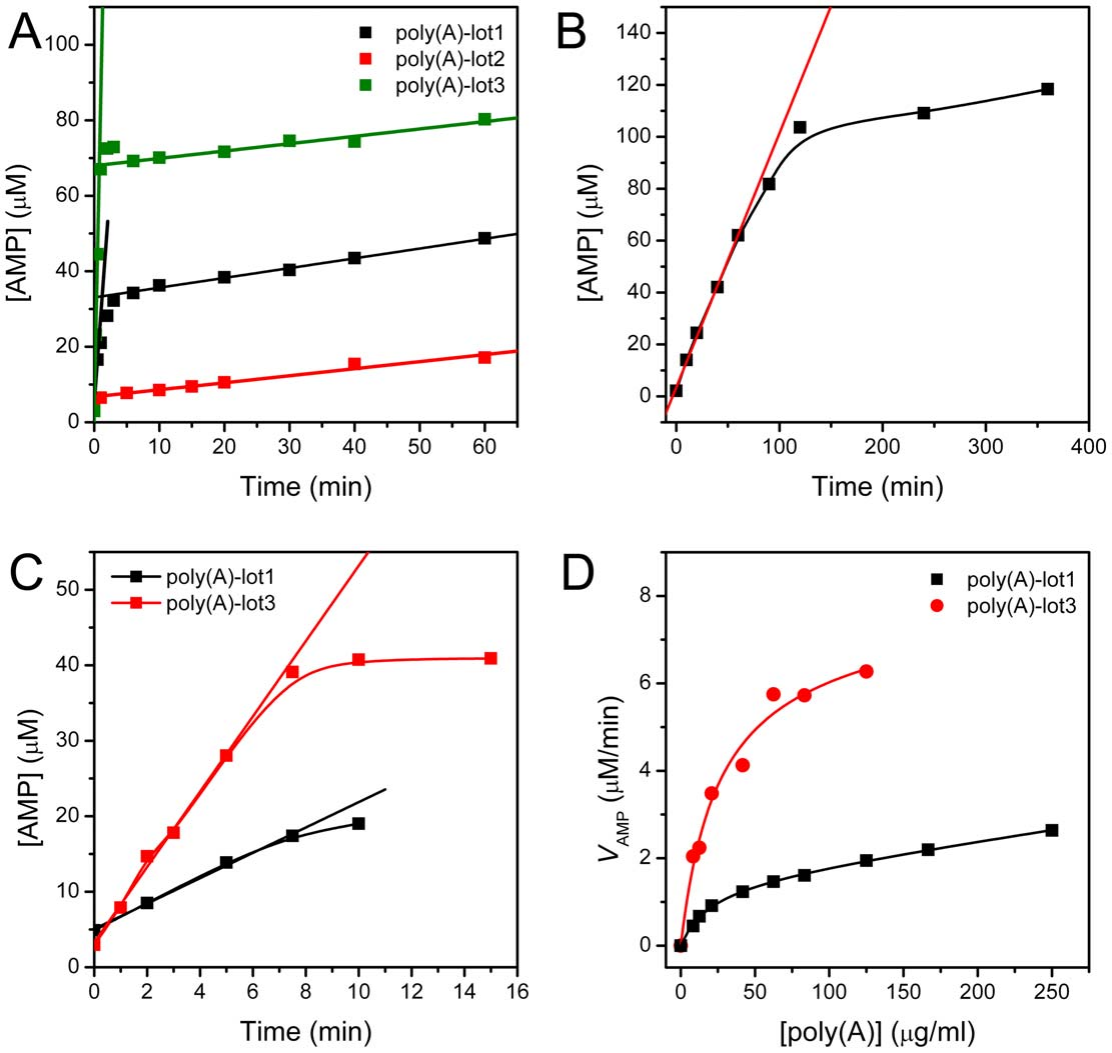
SEC assay of PARN using the three lots of commercial poly(A) as the substrate. (A) Time-course generation of AMP during the enzymatic reaction of PARN using the three lots of commercial poly(A) as the substrate. The enzyme concentration was 0.02 mg/ml. The fast and slow phases of the biphasic process were fitted by linear equation, and the fitted data are showed in solid lines. (B) Time-course generation of AMP when using poly(A)-lot2 as the substrate and a high enzyme concentration of 0.2 mg/ml. (C) The fast phase of AMP generation when using poly(A)-lot1 and poly(A)-lot3 as the substrate. The final concentration of PARN was 0.001 mg/ml. (D) Determination of PARN activity by fitting the experimental data using the Michaelis-Menten kinetics.

The time course SEC profile was recorded and the AMP production vs. time were obtained to reveal the kinetic behavior of PARN catalysis using the commercial poly(A) as the substrate (Figure 5A). Surprisingly, the production of AMP showed an apparent two-phase process when using poly(A)-lot1 and poly(A)-lot3 as the substrates. The duration of fast phase was about 1–2 min at a p60 concentration of 0.02 mg/ml. The slope of fast phase were about 90- and 426- fold larger than that of the slow phase when using poly(A)-lot1 and poly(A)-lot3, respectively. The fast phase was too short to be monitored when using poly(A)-lot2 as the substrate. The slopes of the slow phase of the three kinds of substrates were almost identical, about 0.2 µM/min. The major difference between the three curves was the intersection point of slow phase at the vertical axis, which showed a good agreement with the relative activities of PARN measured using the three lots of substrate. The existence of the slow phase was also evidenced by degradation of poly(A)-lot2 using a high concentration of PARN (0.2 mg/ml), and a typical AMP production curve was observed with a linear part within the first 60 min reaction and a saturation at longer time (Figure 5B). The reason for such a two-phase behavior is unclear. A possible explanation is that the commercial poly(A) existed in two distinct states: one was available for the degradation by PARN, while the other was difficult. The proportions of two states of poly(A) were independent on the size distribution of the sample as revealed in Figures 1 and 4. It is possible that the hardly degraded state was in high-order structures rather than a long random chain, which might prevent the binding of the substrate or cleavage action of PARN. However, this is difficult to elucidate since the high heterogeneity of the commercial poly(A).

Nonetheless, the intersection point was proportional to the concentration of the substrate (data not shown), indicating that the fractions of the fast phase reaction was correlated with the activity measured by either the methylene blue or SEC assay. To ensure the accuracy of the assay, the enzyme concentration and reaction time were screened, and it was found that when the p60 concentration was 0.001 mg/ml, AMP was produced linearly within 8 min reaction (Figure 5C). Under these conditions, the kinetic parameters were determined using poly(A)-lot3 as the substrate (Figure 5D). The *K*_m_-poly(A)-lot3 and *V*_max_-poly(A)-lot3 values were 27±5 µg/ml and 7.7±0.5 µM/min, while the those for poly(A)-lot1 were 20±2 µg/ml and 1.9±0.6 µM/min, respectively. The *K*_m_ values were similar when determined using different lots of the poly(A) substrate, while the *V*_max_ values were significantly different.

It is worth noting that the *V*_max_-poly(A) value of p60 was close to *V*_max_-A20, suggesting that PARN had the same catalytic efficiency at least for oligo(A) larger than 20-mer. Moreover, the *K*_m_-poly(A)-lot3 value was at the same level as that determined by the methylene blue method [27], [31]. These observations might be important for the assay using the commercial poly(A) as the substrate. That is, as long as the major fractions of the commercial poly(A) is longer than 20-mer, the catalytic properties measured by either the methylene blue or SEC assay are convincing. Thus for long poly(A) substrate, the two methods, which are based on the determination of poly(A) shortening or AMP production, are both applicable. The same lot product is recommended when using commercial poly(A) as the substrate for deadenylase assay.

### Pattern of deadenylation characterized by the SEC assay

Deadenylases may degrade the poly(A) tail in a processive or distributive reaction mechanism [14], [20]. According to the criteria to predict a highly processive mode proposed by Martinez et al. [14], in the ideal highly processive mode, the enzyme catalyzed the digestion of the polymeric substrates without dissociation from the substrate until fully deadenylation is achieved. Because A2 is not degraded efficiently in the presence of Mg^2+^ [30], the production of A2 may be similar to that of AMP except that the velocity of A2 production is smaller than that of AMP. The delay time of A2 production is expected to be very short and is dependent on the length of the poly(A) chain. As for the distributive pattern, the deadenylase randomly binds to the 3′-end of the poly(A) molecule and dissociates from the substrate after the removal of one nucleotide. In this case, the substrate molecules are degraded in a synchronous manner and the partially deadenylated substrates will be populated during the reaction.

**Figure 6:**
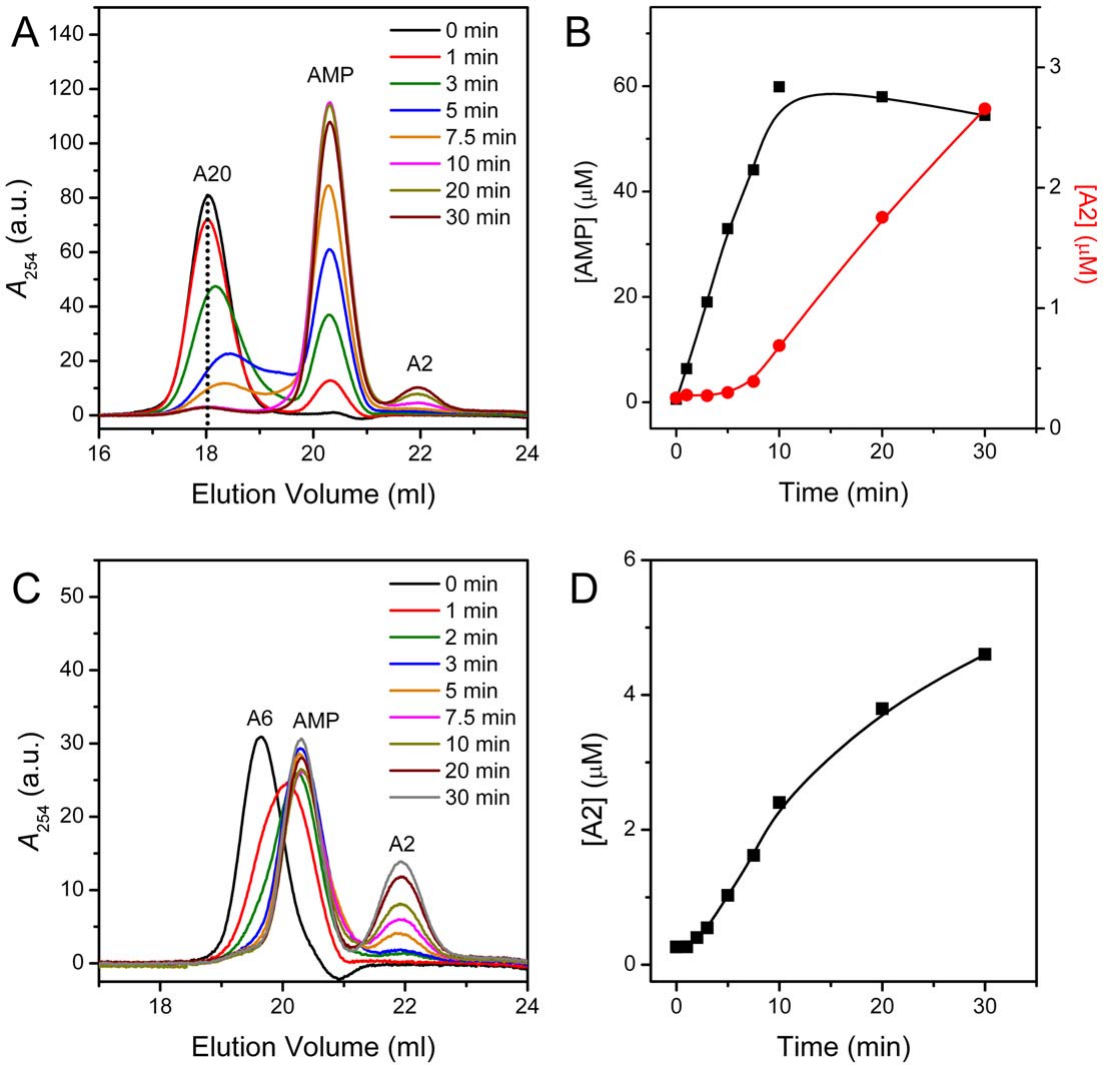
Characterization of the pattern of deadenylation by the SEC assay. (A) SEC elution profiles of the reaction solutions quenched at different time when using 5 µM A20 as the substrate. (B) Kinetics of AMP and A2 generation during A20 degraded by PARN. The concentration of A2 was calculated from the peak area using the standard curve obtained similar to that of AMP shown in Figure 1C. (C) SEC elution profiles of the reaction solutions using 5 µM A6 as the substrate. (D) Kinetics of A2 generation during A6 degraded by PARN. The curve of AMP generation could not be accurately obtained due to the overlapping of the elution peaks from A6 and AMP.

Because AMP and A2 could be well separated by SEC, monitoring the production curves might be able to determine the processive or distributive reaction mechanism of deadenylases. As mentioned above, A2 could hardly be degraded in the presence of Mg^2+^, it could be regarded as the fully deadenylated specie. When 5 µM A20 and 0.001 mg/ml p60 were used in the assay, the concentration of the substrate was more than 300-fold molar excess over that of p60. The time-course AMP and A2 production curves are presented in Figure 6. The kinetic curves indicated that PARN mainly worked in a distributive manner or showed very weak processivity when using the noncapped oligo(A) as the substrate because: i) With the reaction time increased, the elution volume of the substrate shifted to low-molecular-weight position and there was fractions appeared between the peak of A20 and AMP; ii) The amount of AMP increased linearly within 10 min and then remained unchanged, while an about 7.5 min lag time was observed for the production of A2; iii) When A6 was used as the substrate, the AMP curve could not obtained due to peak overlapping problem. Nonetheless, if the elution peaks were separated by curve fitting, the AMP production reached its maximum in about 3 min. The delay time for the generation of A2 was about 1–2 min; iv) The delay time of A2 production for the substrate A20 was 4–5 times longer than that of A6. The conclusion that PARN degraded non-capped poly(A) distributively is consistent with the previous observations that the 5′-cap structure is crucial to the processivity of PARN [13]–[15], [20]. It is worth noting that A2 appeared after A20 or A6 was substantially degraded accompanied with the AMP production close to its maximum. This pronounced long lag time might be caused by that the shorter the substrate, the larger dissociation constants for the enzyme to bind with the substrate smaller than 6-mer [32]. When the substrates were shortened to oligo(A) smaller than 6-mer, the significantly increased dissociation constants delayed the production of A2, and the delay time seems to be proportional to the length of substrates.

### PARN can degrade poly(dA) with low catalytic efficiency

**Figure 7:**
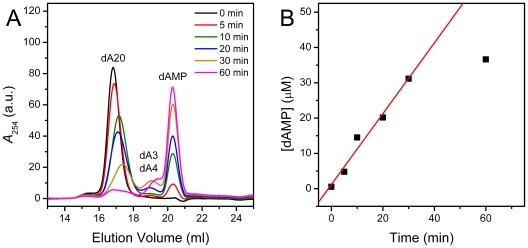
SEC assay of PARN using dA20 as the substrate. (A) The elution profiles of the reaction solutions quenched at different time. The initial dA20 concentration was 2 µM, and the concentration of PARN was 0.1 mg/ml. Details regarding the assay were the same as those in Figure 2. (B) Time-course changes of the dAMP during reaction.

PARN is regarded as a deadenylase with high substrate and metal ion preference [14], [19], [20], and it has been shown that PARN can not degrade DNA [20]. However, when using a relative high enzyme concentration of 0.1 mg/ml, dA20 could be successfully degraded by PARN into dAMP (Figure 7). Similar to the degradation of non-capped A20, dA20 was also cleaved by PARN in a distributive manner as reflected by the peak shift of the substrate along with the reaction time and the accumulation of oligo(dA) intermediate states. The enzymatic parameters were determined by varying the substrate concentration using an enzyme concentration of 0.05 mg/ml. Under these conditions, the *K*_m_-dA20 and *V*_max_-dA20 values of p60 were 35.5 µM and 11.8 µM/min, respectively. The *K*_m_-dA20 vale was ∼130-fold larger than that of *K*_m_-A20, while the *V*_max_-dA20 was ∼35-fold smaller than that of *V*_max_-A20. These observations suggested that PARN could cleave poly(dA) with a dramatically low binding affinity and catalytic efficiency when compared with the degradation of poly(A). It is unclear yet whether the DNA cleavage property of PARN had any physiological relevance. Considering that the full length PARN is located in the nucleus of the cell [20], it is worth investigating whether PARN has additional physiological functions besides acting as a deadenylase.

### The SEC assay is applicable to complex protein mixtures and cell extracts

**Figure 8:**
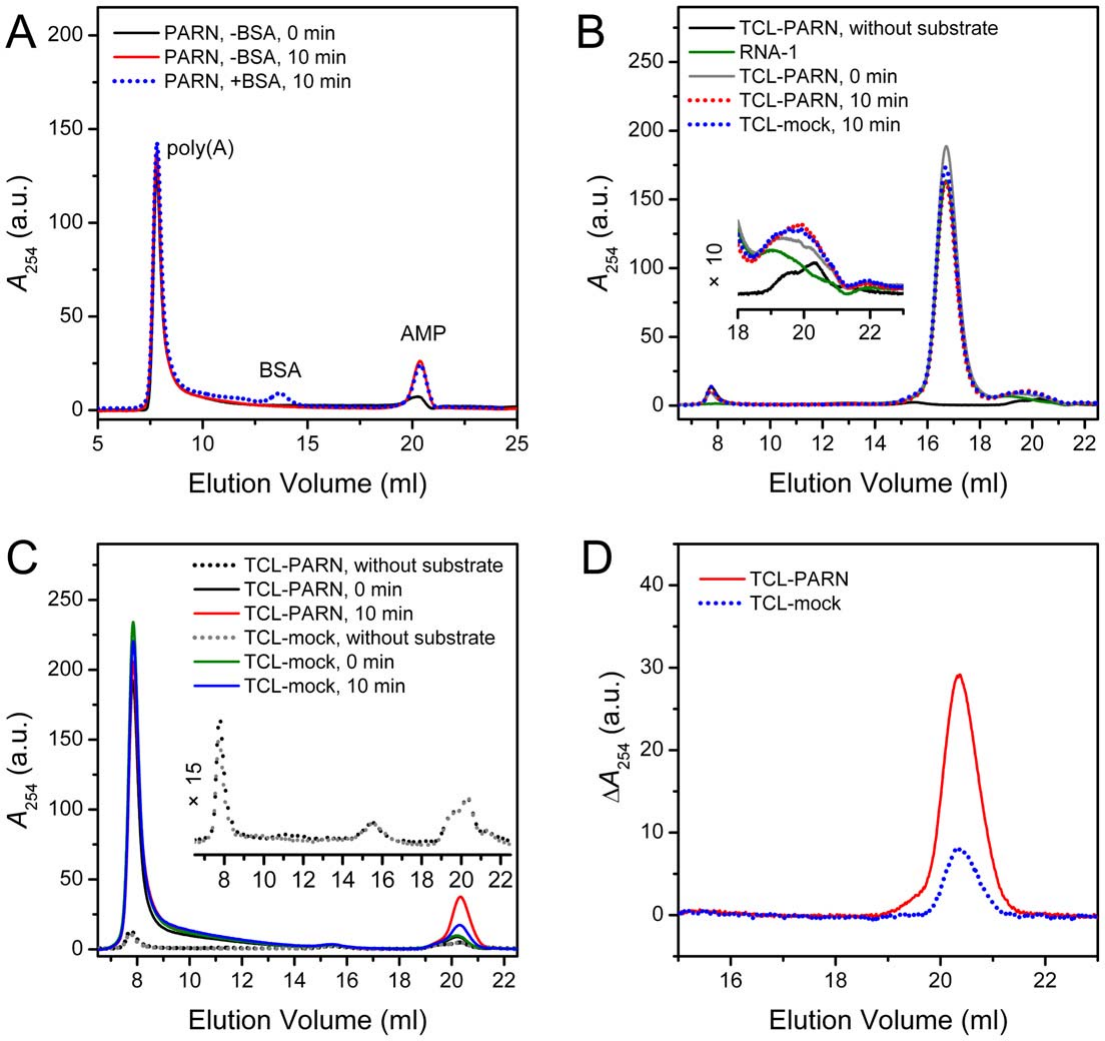
SEC assay of deadenylases in protein mixtures or crude cell extracts. (A) SEC profiles of reaction solutions in the presence or absence of BSA. The final concentrations of PARN, BSA and poly(A)-lot1 were 0.001, 0.2 and 0.1 mg/ml, respectively. The other assay conditions were the same as those in Figure 2. (B) SEC assay of the total cell lysates (TCL, 1∶5 dilution) of HEK-293T cells using a non-poly(A) substrate (designated as RNA-1, 5′-GGAGCUCUGUCCUAUGUAU-3′). The concentration of RNA-1 was 10 µM. The HEK-293T cells were transfected by either the pcDNA3.1 control vector (TCL-mock) or FLAG-PARN (TCL-PARN). The inset shows the high-magnification images of the peaks around the volume where the AMP eluted (20.3 ml). (C) SEC assay of poly(A) degraded by TCL-mock or TCL-PARN. The concentration of the substrate poly(A)-lot1 was 0.1 mg/ml. The inset shows the high-magnification images of SEC profiles of TCL without the addition of the substrate. (D) The difference profiles obtained by subtracting the SEC profiles recorded after 10 min reaction by that at 0 min.

To test whether the SEC assay is also applicable to complex reaction conditions, we tested the assay using protein mixtures and crude cell extracts as the samples. As shown in Figure 8A, when the reaction solution contained 0.2 mg/ml BSA, about 200-fold higher than the concentration of the enzyme PARN, the SEC profile revealed good signal-to-noise ratio for both the substrate and the product with no significant difference with the sample in the absence of BSA. Although BSA has absorption at 254 nm, the influence from the high concentration of proteins could be removed by constructing the difference SEC profiles (data not shown). Moreover, the protein concentration used in the assay is usually much lower than 0.2 mg/ml. For 0.001 mg/ml PARN, no obvious peak could be identified in the SEC profile, suggesting that the SEC assay will not significantly affect by the enzyme or protein mixtures in the reaction solutions. A more complex condition for the assay is the usage of crude cell extracts, which contains various macromolecules and small organic solutes. As presented in Figure 8B, the solutes in the total cell lysates (TCL) only slightly affected the SEC profile of the substrate with a peak eluted at around the void volume and a minor peak appeared at around 15.5 ml. When using a non-poly(A) RNA as the substrate, no significant degradation of the RNA molecule was observed. Considering that PARN is a poly(A)-specific enzyme, the results in Figure 8B indicated that the influence of non-specific ribonucleases on the SEC assay could be neglected under our conditions. The TCL of cells with the transfection of the pcDNA3.1 control vector (TCL-mock) exhibited the deadenylase activity (Figure 8C), which is consistent with the fact that the eukaryotic cells contain several classes of endogenous deadenylases such as PARN, CCR4/CAF1 and Pan2/Pan3 [6], [7]. The observation that the poly(A) but not the non-poly(A) substrate could be degraded by TCL-mock within 10 min reaction might be caused by that deadenylation is the initial step of mRNA decay and mRNA turnover is a fundamental physiological process of the cells. Compared with TCL-mock, the TCL of cells with the overexpression of exogenous PARN had more pronounced ability (∼4 fold) in the degradation of the poly(A) substrate. Similar results could be obtained when using A20 as the substrate (data not shown). The possible interference of the solutes in the TCL could be avoided by the construction of the difference profiles (Figure 8D), which had the same signal-to-noise ratio as the ideal reaction system using the purified recombinant protein.

In conclusion, we found that SEC could be successfully used as a deadenylase assay for both the simple reaction solutions using the purified enzymes and complex conditions such as protein mixtures or crude cell extracts. Since nucleic acid and proteins are distinct in their UV absorbance spectra, the SEC assay could be used for investigating the effects of deadenylase binding partners on deadenylase activity and their association/dissociation in the presence of substrate simultaneously. The SEC assay could also be applied to study the deadenylation pattern of deadenylases by monitoring the status of the substrate and the production of AMP and A2. We also found that the different lots of commercial poly(A) are distinct in their size distributions, structures and activities when determined by the methylene blue or SEC assay. It is suggested that the same lot of commercial sample should be used to ensure the experimental accuracy and reproducibility.
